# Prostate cancer and body size at different ages: an Italian multicentre case–control study

**DOI:** 10.1038/sj.bjc.6601859

**Published:** 2004-05-04

**Authors:** L Dal Maso, A Zucchetto, C La Vecchia, M Montella, E Conti, V Canzonieri, R Talamini, A Tavani, E Negri, A Garbeglio, S Franceschi

**Affiliations:** 1 Servizio di Epidemiologia e Biostatistica, Centro di Riferimento Oncologico, Via Pedemontana Occ.le 12, 33081 Aviano (PN), Italy; 2 Istituto di Ricerche Farmacologiche ‘Mario Negri’, Via Eritrea 62, 20157 Milano, Italy; 3 Istituto di Statistica Medica e Biometria, Università degli Studi di Milano, Via Venezian 1, 20133 Milano, Italy; 4 Servizio di Epidemiologia, Istituto Tumori ‘Fondazione Pascale’, Cappella dei Cangiani, 80131 Napoli, Italy; 5 Servizio Integrato di Epidemiologia e Sistemi Informativi (SINTESI), Via Chianesi 53, 00128 Roma, Italy; 6 Divisione di Anatomia Patologica, Centro di Riferimento Oncologico, Via Pedemontana Occ.le 12, 33081 Aviano (PN), Italy; 7 Unità Operativa di Urologia, Azienda Ospedaliera di Pordenone, Via Montereale 24, 33170 Pordenone, Italy; 8 International Agency for Research on Cancer, 150 Cours A. Thomas, 69008 Lyon, France

**Keywords:** case–control study, prostate cancer, body mass index

## Abstract

We investigated the influence of anthropometric measures at diagnosis and at different ages on prostate cancer risk using an Italian multicentre case–control study conducted between 1991 and 2002 of 1294 histologically confirmed cases and 1451 controls admitted to the same network of hospitals for acute non-neoplastic conditions. Height, weight, body mass index (BMI), waist-to-hip ratio, lean body mass 1 year before diagnosis/interview were not significantly associated with risk. However, a positive association with high BMI at age 30 years was found (odds ratio=1.2 for BMI⩾24.7 *vs* <22.7) and: for less differentiated prostate cancer, with BMI 1 year before diagnosis/interview. This study supports possible relationships between high body mass in young adulthood, and a tendency to high weight throughout adult life, and the risk of prostate cancer.

Age, race and family history are the only well-established risk factors for prostate cancer, one of the commonest types of cancer in developed countries (Hsing and Devesa, 2001; Grönberg, 2003).

Several studies have reported weak or no association (IARC, 2002) with adult weight, body mass index (BMI) and lean body mass (LBM) (Habel *et al*, 2000; Nomura, 2001; Engeland *et al*, 2003; Giles *et al*, 2003). Only a large cohort study (Giovannucci *et al*, 2003b) reported an inverse association with BMI, with a relative risk of 0.5 in younger men. A direct association with body mass measures was found in certain studies focusing on fatal (Andersson *et al*, 1997; Rodriguez *et al*, 2001) or advanced prostate cancer (Putnam *et al*, 2000; Giles *et al*, 2003), suggesting that high BMI may facilitate the progression of prostatic neoplasms (Nomura, 2001).

A moderate positive association between height and prostate cancer was reported in several cohort studies of incidence (Andersson *et al*, 1997; Giovannucci *et al*, 1997; Engeland *et al*, 2003) or mortality (Andersson *et al*, 1997; Freeman *et al*, 2001; Rodriguez *et al*, 2001). Conversely, case–control studies have mainly reported no increase of prostate cancer risk in taller men (Whittemore *et al*, 1995; Villeneuve *et al*, 1999; Gunnell *et al*, 2001; Giles *et al*, 2003).

Waist-to-hip ratio (WHR), an index of central adiposity, was not consistently associated with prostate cancer risk (Whittemore *et al*, 1995; Giles *et al*, 2003; Giovannucci *et al*, 2003b).

Our large Italian case–control study, including extensive information on body size indices at various ages and major potential confounding factors, has allowed further investigation of the role of body size measures in prostate carcinogenesis.

## MATERIALS AND METHODS

Cases included were 1294 men (median age 66, range 46–74 years) with incident, histologically confirmed prostate cancer admitted to the major teaching and general hospitals in the provinces of Pordenone and Gorizia and the greater Milan area in northern Italy, the province of Latina in central Italy and the urban area of Naples in southern Italy.

Controls were 1451 men (median age 63, range 46–74 years), admitted for a wide spectrum of acute conditions unrelated to known or potential risk factors for prostate cancer to hospitals sharing the same catchment's areas of those where cases were referred to. Among controls, 32% had nontraumatic orthopaedic disorders, 21% traumas, 17% surgical conditions and 29% miscellaneous other illnesses, such as eye, ear and dental disorders. Less than 5% of both cases and controls contacted refused the interview, and the participation did not vary across hospitals and geographic areas.

All interviews were conducted in a hospital setting using a structured questionnaire, which included information on age, education and other socioeconomic factors, physical activity, smoking habit, alcohol intake, an itemised food frequency section, a problem-oriented medical history and history of cancer in first-degree relatives. Body size indexes at different ages were collected in a detailed section of the questionnaire. Study subjects were asked to report their height and habitual weight 1 year prior to cancer diagnosis or interview (in controls). Information on perceived body size at 12 years of age (i.e.: thinner than, same as, heavier than peers), weight at ages 30 and 50, highest and lowest weight in adult life were also collected. The interviewers measured the circumference of the waist (2 cm above the umbilicus) and hips (maximal protrusion), and WHR was computed. In 25% of prostate cancer cases and 24% of control subjects, waist or hip could not be measured for technical reasons. BMI was computed as weight/height^2^ (kg m^−2^) and, since it was suggested that BMI does not differentiate lean and fat masses (Nomura, 2001), LBM was also computed using the appropriate algorithm ((2.447−0.09516 × age+0.1074 × height+0.3362 × weight)/0.732) (Willett, 1999).

Approximate tertiles or quartiles by various body size indexes were computed on the basis of the combined distribution of cases and controls. Odds ratios (OR) of prostate cancer and the corresponding 95% confidence intervals (CI), for various body measures, were calculated using unconditional multiple logistic regression, fitted by the method of maximum likelihood (Breslow and Day, 1980). The effect of several potential confounding factors was considered, including study centre, age in 5-year groups, education, occupational physical activity and family history of prostate cancer in first-degree relatives. Additional adjustment for energy intake and smoking habits did not materially modify our results. Tests for linear trends were assessed by means of the Wald *χ*^2^ on the variables considered as categorical. Selected analyses were repeated separately according to the TNM pathological stage (Sobin and Wittekind, 1997) and the degree of histological differentiation (Gleason score; Gleason and Mellinger, 1974).

## RESULTS

Table 1

**Table 1:** Distribution of 1294 cases of prostate cancer and 1451 controls according to age and selected covariates. Italy, 1991–2002

	**Cases no. (%)**	**Controls no. (%)**
*Age*		
45–59	219 (16.9)	431 (29.7)
60–64	310 (24.0)	359 (24.7)
65–69	419 (32.4)	364 (25.1)
70–74	346 (26.7)	297 (20.5)
		
*Education (years)*^a^		
<7	636 (49.6)	844 (58.5)
7–11	384 (29.9)	407 (28.2)
⩾12	263 (20.5)	192 (13.3)
		
*Occupational physical activity at age 30*^a^		
Very active	518 (40.2)	684 (47.1)
Moderately active	335 (26.0)	393 (27.1)
Inactive	437 (33.9)	374 (25.8)
		
*Family history of prostate cancer in first-degree relatives*		
No	1204 (93.0)	1423 (98.1)
Yes	90 (7.0)	28 (1.9)
		
*Pathological stage (TNM)*		
I–II	321 (24.8)	
III–IV	231 (17.9)	
Unknown	742 (57.3)	
		
*Histological grade (Gleason score)*		
2–6 (more differentiated)	538 (41.6)	
7–10	384 (29.7)	
Unknown	372 (28.8)	

a The sum does not add up to the total because of some missing values.

gives the distribution of cases and controls according to age and selected covariates. Prostate cancer cases were significantly more educated than controls; they had a lower occupational physical activity, and reported a family history of prostate cancer (7%) more frequently than controls (2%). Pathological stage was available in 42% of prostate cancer cases and Gleason score in 71% (Table 1).

Table 2

**Table 2:** Distribution of 1294 cases of prostate cancer and 1451 controls, OR and corresponding 95% CI, according to body-size measures at diagnosis/interview. Italy, 1991–2002

	**Cases**	**Controls**	**OR**	**(95% CI)**
*Height (cm)*				
<169	353	420	1	
169–172	353	372	1.11	(0.89–1.37)
173–176	278	323	0.96	(0.76–1.20)
⩾177	307	335	0.98	(0.78–1.23)
*χ*^2^ trend			0.30	*P*=0.59
				
*Weight (kg)*				
<71	303	370	1	
71–78	359	374	1.13	(0.91–1.40)
79–85	343	356	1.16	(0.93–1.45)
⩾86	287	348	1.02	(0.81–1.28)
*χ*^2^ trend			0.05	*P*=0.82
				
*Body mass index (kg m*^−*2*^)				
<24.22	301	368	1	
24.22 to <26.18	346	356	1.18	(0.95–1.47)
26.18 to <28.41	324	365	1.12	(0.89–1.40)
⩾28.41	319	358	1.18	(0.94–1.47)
*χ*^2^ trend			1.42	*P*=0.23
				
*Waist-to-hip ratio*				
<0.93	229	240	1	
0.93 to <0.96	209	293	0.74	(0.57–0.97)
0.96 to < 0.99	270	296	0.93	(0.72–1.19)
⩾0.99	258	279	0.95	(0.73–1.24)
*χ*^2^ trend			0.01	*P*=0.93
				
*Lean body mass*				
<52.36	315	368	1	
52.36 to <56.19	341	346	1.17	(0.94–1.46)
56.19 to <59.88	321	362	1.10	(0.88–1.38)
⩾59.88	313	371	1.09	(0.87–1.37)
*χ*^2^ trend			0.28	*P*=0.60

a Estimates from multiple logistic regression equations, including terms for age (quinquennia), study centre, education, physical activity and family history of prostate cancer.

b The sum may not add up to the total because of some missing values.

shows the distribution of prostate cancer and controls and the corresponding ORs, according to various body size measures 1 year before diagnosis/interview. Comparing the highest with the lowest quartiles, the ORs were close to unity for height, weight, WHR and LBM. The three highest quartiles of BMI were associated with slightly increased ORs, compared to the lowest quartile, but the risk trend was not significant (*P*=0.23).

No association with prostate cancer risk was found with perceived body size compared to peers at 12 years of age (Table 3

**Table 3:** Distribution of 1294 cases of prostate cancer and 1451 controls, OR and corresponding 95% CI, according to body-size measures during lifetime. Italy, 1991–2002

	**Cases**	**Controls**	**OR**	**(95% CI)**
*Perceived body size at age 12*				
Thinner	521	575	1	
Same	492	549	1.00	(0.84–1.20)
Heavier	265	315	0.95	(0.77–1.17)
*χ*^2^ trend			0.18	*P*=0.67
				
*BMI at age 30 (kg m*^−*2*^)				
<22.65	406	492	1	
22.65 to <24.69	437	430	1.33	(1.09–1.62)
⩾24.69	414	459	1.22	(1.01–1.48)
*χ*^2^ trend			4.04	*P*=0.04
				
*Lowest BMI (kg m*^−*2*^)				
<21.22	420	490	1	
21.22 to <23.14	402	468	1.08	(0.89–1.31)
⩾23.14	447	443	1.32	(1.08–1.60)
*χ*^2^ trend			7.70	*P*<0.01
				
*Increase of BMI from lowest (kg m*^−*2*^)^c^				
<3.15	412	412	1	
3.15 to <5.67	399	402	0.98	(0.80–1.20)
⩾5.67	359	442	0.82	(0.67–1.01)
*χ*^2^ trend			3.54	*P*=0.06

c Subject with lowest BMI reported less than 5 years before diagnosis/interview were excluded.

a Estimates from multiple logistic regression equations, including terms for age (quinquennia), study centre, education, physical activity and family history of prostate cancer.

b The sum may not add up to the total because of some missing values.

). However, a weak direct association was found with BMI at age 30 (OR=1.2; 95% CI: 1.0–1.5, for BMI⩾24.7 *vs* <22.7), and with the lowest BMI in adult life (OR=1.3, 95% CI: 1.1–1.6, for BMI⩾23.1 *vs* <21.2). Subjects with lifetime BMI increase equal to 5.7 units or greater, compared to the lowest tertile (less than 3.2): had an OR of developing prostate cancer of 0.8 (95% CI: 0.7–1.0) (Table 3).

The pattern of association of prostate cancer with BMI at diagnosis/interview or at age 30 was similar below and above 65 years of age at diagnosis. The association between LBM at various ages and prostate cancer risk was similar to that for BMI; no association emerged with BMI at age 50 and maximal lifetime BMI (data not shown in tables).

Table 4

**Table 4:** Distribution of prostate cancer cases and controls, OR and corresponding 95% CI according to stage, grade, and BMI (kg m^−2^) at diagnosis/interview and at age 30. Italy, 1991–2002

		**Stage (TNM)**				**Grade (Gleason score)**			
		**I–II**		**III–IV**		**2–6**		**7–10**	
	**Controls**	**Cases**	**OR (95% CI)**	**Cases**	**OR (95% CI)**	**Cases**	**OR (95% CI)**	**Cases**	**OR (95% CI)**
*BMI at diagnosis/interview*									
<24.22	368	79	1	45	1	126	1	69	1
24.22 to <26.18	356	88	1.12 (0.78–1.60)	67	1.47 (0.96–2.25)	153	1.27 (0.95–1.69)	102	1.49 (1.05–2.12)
26.18 to <28.41	365	77	0.91 (0.63–1.32)	59	1.27 (0.83–1.96)	125	0.98 (0.72–1.32)	111	1.57 (1.11–2.22)
⩾28.41	358	76	1.02 (0.70–1.47)	60	1.40 (0.91–2.16)	134	1.14 (0.85–1.53)	102	1.61 (1.13–2.28)
*χ*^2^ trend		0.07	*P*=0.79	1.38	*P*=0.24	0.06	*P*=0.81	6.38	*P*=0.01
									
*BMI at age 30*									
<22.65	492	102	1	59	1	157	1	112	1
22.65 to <24.69	430	109	1.21 (0.87–1.67)	88	1.87 (1.29–2.72)	190	1.50 (1.16–1.95)	139	1.60 (1.19–2.15)
⩾24.69	459	107	1.20 (0.87–1.65)	84	1.62 (1.11–2.35)	180	1.36 (1.04–1.76)	129	1.39 (1.03–1.87)
*χ*^2^ trend		1.25	*P*=0.26	5.79	*P*=0.02	5.12	*P*=0.02	4.64	*P*=0.03

a Estimates from multiple logistic regression equations, including terms for age (quinquennia), study centre, education, physical activity, and family history of prostate cancer.

shows the association of prostate cancer and BMI at diagnosis/interview and at age 30 in different strata of pathological stage and Gleason score. The direct association with BMI at diagnosis/interview was slightly stronger among men with stage III–IV and less differentiated tumours. The OR for BMI ⩾28.4 *vs* <24.2 among cases with Gleason score 7–10 was 1.6 (95% CI: 1.1–2.3). The association with BMI at age 30 was consistent in different strata of pathological stage and Gleason score (Table 4).

## DISCUSSION

The main findings of our study are associations between prostate cancer and BMI at age 30 years and with a relatively elevated BMI throughout adult life, as implied by reporting a relatively high lowest BMI or a low BMI increase life long (Table 3). No trend in risk according to BMI at diagnosis/interview emerged. Weight, WHR and LBM at ages close to diagnosis or during adolescence were also unrelated to prostate cancer risk.

Some studies (Andersson *et al*, 1997; Giovannucci *et al*, 1997) have shown a direct relationship between height and prostate cancer risk, suggesting a possible role of nutritional status or levels of circulating growth factors during puberty. Height, however, was unrelated to prostate cancer risk in our study and in several other studies (Hsieh *et al*, 1999; Hsing *et al*, 2000; Rodriguez *et al*, 2001).

With few exceptions, recent BMI was also found to be unrelated to prostate cancer risk in case–control studies (Kolonel, 1996; Giles *et al*, 2003). Prospective studies are more supportive of a positive association, particularly, those that included prostate cancer mortality as end point (Putnam *et al*, 2000; Rodriguez *et al*, 2001). Obesity and its hormonal and metabolic correlates may increase prostate cancer progression and decrease survival. In our study, cases with less differentiated and, hence, prognostically worse prostate cancer (Gleason and Mellinger, 1974) showed an association with BMI at diagnosis, although the findings by Gleason score were not significantly heterogeneous. Moreover, a positive modest association between measures of adiposity and the risk of aggressive disease emerged also in a prospective cohort study from Australia (MacInnis *et al*, 2003).

The relevant exposure period for the association between body size and prostate cancer, if it exists, is also unclear. Being overweight in adolescence (i.e. perceived body size compared to one's peers at age 12) was unrelated to prostate cancer risk in our as well as in a few previous studies (Giovannucci *et al*, 1997; Giles *et al*, 2003). We found, however, a direct association with weight gain early in adult life and, notably, with BMI at age 30 years. It is possible that, conversely to breast cancer in women, which is greatly affected by events in puberty and young adulthood, the most relevant exposure period for prostate cancer may be later, perhaps in the fourth decade of life. This may be indirectly supported by the special age distribution of prostate cancer incidence that shows an exponential rise beginning at approximately age 50–55 (i.e. later than any other cancer site, including hormone dependent tumours in women) (Signorello and Adami, 2002). The association between weight or BMI and prostate cancer must be, however, weak or, possibly, weakened by some ill-understood heterogeneity in the disease.

Effects of long-term, even mild, overweight on prostate cancer risk could be mediated by several biological mechanisms, implicating sex hormones (androgens and oestrogen) (Nomura, 2001), leptin, and insulin-like growth factor (IGF)-I (Chan *et al*, 1998; Calle, 2000; Hsing and Devesa, 2001; Pollak, 2001). However, the relationship between nutritional factors, IGF system, serum and tissue hormones, anthropometric measures, and prostate carcinogenesis is complex and ill understood (Calle, 2000; Pollak, 2001; Moyad, 2002; Giovannucci *et al*, 2003a).

In our study, problems of reliability in anthropometric measures cannot be excluded; however, there is no evidence that weight was differentially reported by cases and controls (Casey *et al*, 1991). Indeed, there was no reason for recall bias in current weight and height, in our study, as cases and controls were interviewed in similar hospital settings and the general population was unaware of the possible relationship (D'Avanzo *et al*, 1997). Past body measures are generally well correlated with corresponding measures even in older persons (Klipstein-Grobusch *et al*, 1998).

Hospital-based case–control studies may be more susceptible to selection and information bias than cohort studies (Breslow and Day, 1980). Cases and controls in our study, however, were selected from the same catchment's areas, and participation rate was equally high. Subjects with diseases potentially linked to diet and dietary modifications were excluded from the control group, and major confounding factors of prostate cancer were adjusted for. In particular, careful allowance was made for education and social class, which were directly related to prostate cancer risk, and may go along with a different prevalence of PSA testing.

In conclusion, this uniquely large case–control study on prostate cancer, conducted in a southern European population, showed no strong role for a wide range of anthropometric measures at various ages. However, weight gain early in adulthood seems to be a risk factor and supports a role for hormonal or metabolic correlates of overweight in the onset or progression of prostate cancers.
